# Assessment of iodine importance and needs for supplementation in school-aged children in Portugal

**DOI:** 10.1186/s40795-017-0175-x

**Published:** 2017-07-20

**Authors:** Ana M. Pires, Sandra Félix, Ana C. C. Sousa

**Affiliations:** 1 Universidade Atlântica – Centro de Estudos, Sociedade, Organizações e Bem-Estar (CESOB), Oeiras, Portugal; 2 Faculdade de Ciências da Universidade de Lisboa – Centre for Ecology, Evolution and Environmental Changes (Ce3C), Lisbon, Portugal

**Keywords:** Iodine, Supplementation, Promotion, Health protection

## Abstract

**Background:**

Micronutrients are essential for child proper growth and development. Nutritional deficiencies of these elements have increasingly been a concern in Europe, as they are often related to the cognitive potential and physical lifelong consequences. However, being an essential trace element for thyroid function, iodine intake in the desired quantities becomes also very important for neurodevelopment, including for school-aged children. Therefore, the aim of this paper is to highlight the need for wider dissemination of the physiological importance of iodine among health professionals and the general population as well as the knowledge of iodine needs and possible supplementation within families with school-aged children.

**Methods:**

The present study is an observational, descriptive, cross-sectional evaluation of knowledge and perceptions of iodine physiological importance. An evaluation survey has been carried out based on knowledge of iodine needs and possible supplementation within families with school-aged children. It has been target at mothers with school-age children under 18 years old with residence in Portugal. Data are represented as frequency and percentages and association between variables was measured.

**Results:**

The internet survey has been answered by around 691 mothers, corresponding to 811 children data validated; 47% (*n* = 381) girls and 53% (*n* = 430) boys. Mother’s knowledge about iodine and the importance for the improvement of learning capacity is not independent of having health study/work area (χ^2^ at the 0.05 level). Nevertheless, it can be observed a slight association between mothers who agree with iodine supplementation and those who effectively supplement their child (χ^2^ 9.315; Φ 0.116). Although use of iodized kitchen salt certainly changes the balance from sub-optimal to adequate iodine nutrition, only 8.8% (*n* = 61) reported using iodized salt. However, 87.6% (*n* = 605) assumed salt iodization importance without information.

**Conclusions:**

We believe that the findings of this survey have great public health importance for Portugal. While many countries have mandatory iodizes salt programmes, in Portugal iodine supplementation is not a current practice. Therefore, we suggest an urgent evaluation of iodine in Portugal, namely for school-aged children, where iodine deficiencies are critical, as well as a systematic information dissemination as a form of publicizing iodine supplementation needs.

**Electronic supplementary material:**

The online version of this article (doi:10.1186/s40795-017-0175-x) contains supplementary material, which is available to authorized users.

## Background

The public health and nutrition policy in Europe is focused largely on addressing problems of over-consumption [1, 2]. Yet, even in the midst of an abundant dietary supply, important questions emerge regarding the prevalence of suboptimal micronutrients intakes [3].

Children and adolescents require higher ratio of nutrients by body weight than adults, not only because of a higher metabolic rate, but also because vitamin and mineral intakes tend to be reduced due to a decreased vegetable and fruit intake [4, 5]. Population-based data suggests substantial variability in micronutrients intake among children in Europe and data highlight common deficiencies for several micronutrients, such as iodine [6–12].

Iodine is an essential trace element for thyroid hormones synthesis, and dietary intakes of this mineral is necessary for its production. Thyroid hormones regulate the metabolic pattern of most cells of the organism, and they are essential for a correct brain and development of cognitive potential. Although cretinism is the most extreme manifestation resulting from congenital deficiency of these hormones, the more subtle degrees of mental impairment leading to poor school performance, reduced intellectual ability and impaired work capacity are of considerably greater significance [13–16].

Several studies showed the connection between iodine proper intake and cognitive development [17, 18] and a link between iodine deficiency and attention deficit disorders [19]. Also Delange [20], Santiago-Fernandez et al. [21] and EFSA Panel [22] associated iodine deficiency with a global loss of 10–15 IQ points at a population level and refer it as the world’s greatest single cause of preventable brain damage and mental retardation.

Due to the essential role of iodine during embryonic development, supplementation in pregnancy is widespread although an inadequate iodine supply in pregnancy is present in approximately 30% of European countries [7]. For all these reasons, the World Health Organization (WHO) [23], recommended school-age-children (SAC) and pregnant women as being the indicator groups to evaluate iodine sufficiency.

Since iodine deficiency has emerged as a public health issue following several decades of apparent iodine sufficiency, major progresses have been made from 2003 to 2011, after the WHO alert for iodine importance [23, 24], making the number of iodine-deficient countries decreased. However, worldwide, 29,8% of SAC (≈ 246 million) are estimated to have insufficient iodine intakes [13, 25–27], whereas the greatest proportions of SAC with inadequate iodine intake are in Europe (43,9%) [27, 28]. Iodized salt programs [29], have shown to be effective and WHO does not recommended other iodine supplementation [23].

### Portuguese situation

Studies in Portugal on pregnant women point out to a generalized insufficient iodine intake all over the country [30, 31] with differences from pregnant women by the sea and the hinterland, being the reduced fish consumption and the lower socioeconomic status the justification for those differences. However, the islands of Açores and Madeira values were even lower than those obtained in the hinterland continental Portugal [31].

Although in 2007, the WHO [23] included Portugal in the group of countries where iodine supplementation of pregnant women and infants is recommend, the Portuguese Directorate-General for Health (DGS) recommendation for iodine supplementation emerged only in august 2013 for pregnancy and breastfeeding women [32]. However, there is little available information about monitoring of iodine levels, even in the population of pregnant and lactating women, was not performed [33].

While other studies already suggest monitoring of the ongoing programs [34], no current data have been considered for SAC Portuguese population, in international studies [6, 7, 26, 27]. The only available data for children in Portugal are those from [35], were 47% of children aged 6–12 years show an inadequate iodine intake, being islands values even lower [36], which according Vanderpump et al. [28] is confirming that the proximity of the sea does not prevent iodine deficiency.

Therefore, the aim of this paper is to highlight the need for wider dissemination of the physiological importance of iodine among health professionals and the general population. In this sense, an evaluation survey has been carried out on the knowledge of iodine needs and possible supplementation within families with school-aged children.

## Methods

The present study is an observational, descriptive, cross-sectional evaluation of knowledge and perceptions of iodine physiological importance, based on survey research methodology. The development and implementation of an online survey questionnaire followed the guidelines for this research methodology [37–39]. The questions were ordered to establish both the survey’s logic and inner flow, and to establish an adequate time needed for each answer. The questionnaire included three sections with information about mothers’ characterization (1^st^ section: age, living area, academic qualifications, work or study in health area, how many children have with ≤18 years old); children’s characterization and their routines ( 2^nd^ section: gender, age, lunch at school canteen, if mother’s took their children to pediatrician at least once a year and if yes, if talk about feeding habits, any chronic disease, any food intolerance) and eating habits at home and knowledge about iodine (3^rd^ section: any special attention to diet during pregnancy, any supplementation during pregnancy, knowledge about the amount of iodine needed during pregnancy, if usually gave any supplementation to children, which salt type(s) used at home (considering all salt types available on market, with or without iodine added: sea salt; rock salt; iodized salt), the knowledge about iodine being a mineral or other food group, function of iodine, if agree/disagree or have no information/interest about iodine supplementation, interested in more information on this and other nutrients). Also included in this 3^rd^ section, mother’s answered to an adapted food frequency questionnaire about children feeding habits, and identify whether or not iodine is present in some types of foods (annex 1). All questions of the questionnaire were closed-ended, allowing one option (or more than one in a few questions such us “*which salt type(s) used at home*”). These questions are addressed in the results tables and questionnaire is available on https://figshare.com/account/articles/5135266 (Additional file 1). The responses were previously validated by three medical doctors, having been unanimous in highlighting the relevance of the study and also the proposed questions.

A widespread dissemination was done by email, containing a link which allowed easy access to the survey, for mailing lists and through social networks, ensuring a cascaded reach. Data collection took place between March 1 and April 15, 2016. It has been target at mothers with school-age children under 18 years old with residence in Portugal, mainland and islands.

The validity of the data were well established since the pattern of data remained constant from half of the questionaries’ responses.

Data are represented as frequency and percentages. Statistical analysis was done using SPSS software (Version 21; SPSS, Inc., Chicago, IL). Association between variables was measured by Chi-square test (χ^2^), once data were qualitatively collected. The strength of association between variables was measured by calculating Phi (Φ) values (at 0.05 level).

## Results

The internet survey focused on several factors (Table 1), it was disseminated through random contacts and has been answered by around 691 mothers, corresponding to 811 children data validated; 47% (*n* = 381) girls and 53% (*n* = 430) boys. Mothers aged from 31 to 40 years (46.7%) and 41 to 50 years (42.2%) reached almost 90% of the all the sample (median 39.3; mode 38.7). Although the majority of mothers sampled were from the Lisbon region (51.4%), all the defined regions across the country were represented and 76.6% of them had a high education level (Table 2).

**Table 1:** Factors evaluated within the sample survey

**Mothers**: * Age* * Living area* * Academic Qualifications* * Study/work in health or other area* * Take children to pediatrician and talk about feeding habits…* * Special attention to diet during pregnancy…* * Supplementation during pregnancy…* * Salt type used…* * Knowledge about iodine…* * Function of iodine…* * Food groups rich in iodine…* * Regarding iodine supplementation…* * Interest in more information on this and other nutrients…*
**Children**: * Sex* * Age* * Lunch at school canteen…* * Any supplementation…* * Food frequency…*

**Table 2:** Mothers’ data characterization (*n* = 691)

	Total (*n* = 691)	Health study/work area (*n* = 107; 15.5%)	Others study/work area (*n* = 584; 84.5%)	χ^2^	Φ
*Knowledge about iodine…*	Correct = 516 (74.7%) Incorrect = 41 (5.9%) Do not know = 134 (19.4%)	Correct = *n* = 97 (90.6%) Incorrect = 2 (1.9%) Do not know = 8 (7.5%)	Correct = 419 (71.7%) Incorrect = 39 (6.7%) Do not know = 126 (21.6%)	17.095	0.157
*Function of iodine…*	Correct = 121 (17.5%) Incorrect = 348 (50.4%) Do not know = 570 (32.1%)	Correct = 35 (32.7%) Incorrect = 50 (46.7%) Do not know = 22 (20.6%)	Correct = 86 (14.7%) Incorrect = 298 (51.0%) Do not know = 200 (34.3%)	20.249	0.171
*Iodine supplementation…*	Yes = 110 (15.9%) No = 8 (1.2%) Do not know = 573 (82.9%)	Yes = 22 (20.6%) No = 0 (0.0%) Do not know = 85 (79.4%)	Yes = 88 (15%) No = 8 (1.4%) Do not know = 488 (83.6%)	2.038	0.054

Mother’s knowledge about iodine being a mineral and the importance for the improvement of learning capacity is not independent of having health study/work area (χ^2^at the 0.05 level). Although Φ values are not significant, percentages of correct answers are greater in health study/work mothers group. On the other hand, the knowledge about the needs of iodine supplementation is independent of having health study/work area. Nevertheless, it can be observed a slight association between mothers who agree with iodine supplementation and those who effectively supplement their child (χ^2^ 9.315; Φ 0.116).

As iodine being suggested as a supplement for pregnancy and lactation periods [32], a separated analysis has been done using data from mothers with children under 3 years old. Analyzing those data (Table 3), only 16 of 120 were supplemented with iodine, five of which agreed with supplementation and 11 have no opinion or do not know. Curiously, although 33 of these mothers study or work in health field, only five of them agree with supplementation, but they were not supplemented with iodine. On the other hand, four of those iodine supplemented mothers answered as do “*not have enough information to decide*”.

**Table 3:** Mothers’ data with children aged <3 years (*n* = 120; 17.4% of total mothers’ sample)

	*n* = 120
*Take iodine supplementation during pregnancy…*	16 (13.3%)
*Take children to the pediatrician and talk about feeding habits…*	113 (94.2%)
*Give supplements to children…*	16 (13.3%)
*Iodized salt using…*	11 (9.2%)
*Knowledge about iodine…*	Correct = 95 (79.2%) Incorrect = 3 (2.5%) Do not know = 22 (18.3%)
*Function of iodine…*	Correct = 27 (22.5%) Incorrect = 59 (49.2%) Do not know = 34 (28.3%)

All children five aged groups evaluated (<3 years; 4 to 6 years, 7 to 9 years, 10 to 14 years and 15 to 18 years) were similarly represented, being the age group 10 to 14 years more representative (30.5%) (Table 4).

**Table 4:** Children sample data characterization (*n* = 811)

Data	Total Sample (*n* = 811)	<3 years (*n* = 144; 17.8%)	4–6 years (*n* = 134; 16.5%)	7–9 years (*n* = 160; 19.7%)	10–14 years (*n* = 247; 30.5%)	15–18 years (*n* = 126; 15.5%)
Sex						
Male	*n* = 430 (53.0%)	*n* = 68 (47.3%)	*n* = 77 (57.5%)	*n* = 77 (48.1%)	*n* = 141 (57.1%)	*n* = 67 (53.2%)
Female	*n* = 381 (47.0%)	*n* = 76 (52.7%)	*n* = 57 (42.5%)	*n* = 83 (51.9%)	*n* = 106 (42.9%)	*n* = 59 (46.8%)
*Any food supplement…*	*n* = 102 (12.6%)	*n* = 21 (20.6%)	*n* = 14 (13.7%)	*n* = 18 (17.6%)	*n* = 37 (36.3%)	*n* = 12 (11.8%)
*Lunch at school cantineen…*	*n* = 560 (69.1%)	*n* = 97 (67.4%)	*n* = 121 (90.3%)	*n* = 136 (85.0%)	*n* = 160 (64.8%)	*n* = 46 (36.5%)

Concerning the association between child age group and lunch at school canteen, χ^2^ (151.867) and Φ (0.433) values are significant (at 0.05 level), showing that, excluding the youngest age-group (< 3 years old), there is a decrease of children attending school canteen.

Despite considering not having enough information (*n* = 605; 87.6%) about nutrients, the greatest majority of sampled mothers identifies these three groups (Table 5) correctly, as being the most iodine-rich sources (*n* = 412; 59.6%). However, children frequency food table (Table 6) indicates that fish is the most frequent eating group (2–4 times per week) and that algae are almost never included in children food habits.

**Table 5:** Food samples more rich in iodine (*n* = 691)

	Yes	No	Do not know
*Fish*	*n* = 517 (74.8%)	*n* = 23 (3.3%)	*n* = 151 (21.9%)
*Seafood*	*n* = 513 (74.2%)	*n* = 15 (2.2%)	*n* = 163 (23.6%)
*Algae*	*n* = 507 (73.4%)	*n* = 13 (1.9%)	*n* = 171 (24.7%)

**Table 6:**
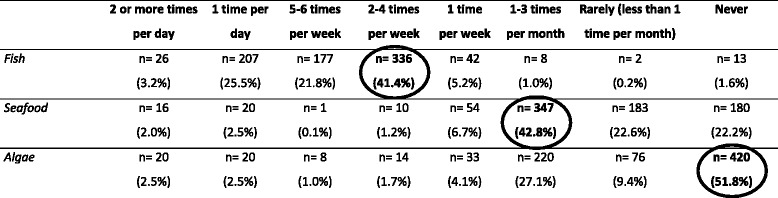
Children eating frequency of food rich in iodine (*n* = 811)

The values corresponding to rich iodized groups are encircled in order to highlight their intake frequency

Although complementary use of iodized kitchen salt certainly changes the balance from sub-optimal to adequate iodine nutrition, only 8.8% (*n* = 61) reported using iodized salt while 91.2% (*n* = 630) use any other salt type. However, 87.6% (*n* = 605) assumed salt iodization importance without information.

## Discussion

Roman-Viñas et al. [40] conclude that several micronutrients, iodine included, showed a high prevalence of inadequate intakes (above 20%) in Europe. This overwhelming evidence makes prevention of iodine deficiency a high global priority to foster children’s development [10]. Despite the global prevalence in School-Aged Children (SAC) of low intakes has fallen over the past 8 years, Europe still shows a higher proportion of children with inadequate iodine intake (43.9%) [26].

Most of iodine comes from feeding. Fish and shellfish are generally a good source, because the ocean contains a considerable amount of iodine. On the contrary, plants which grow in iodine-deficient soils are poor in this element, as well as meat and other animal products feed in plants low in iodine [41, 42]. If iodine intakes are chronically low, thyroidal iodine reserves will be gradually depleted and iodine turnover will need to be increasingly covered by dietary iodine supply [43]. Although food frequency was not analyzed as an objective, also results of the present study corroborate that, despite the knowledge of iodine-rich food groups, these are not frequently included in children meals.

We believe that the findings of this survey have great public health importance for Portugal. While many countries have mandatory iodizes salt programmes in Portugal only two recommendations are ongoing; DGS suggests supplementation for pregnant women [32] and Directorate-General for Education (DGE) the iodized salt use for school canteens [44]. However, although Universal salt iodine can eliminate specific supplementation [45], such as pregnancy and lactation periods [32, 35], our results showed that iodine supplementation is not a current practice. Obstetricians and pediatrics medical practice and mothers did not present consistent information regarding this subject. Considering international knowledge and DGS guidelines [32], it was supposed that mothers with children aged <3 years should have more information regarding iodine needs. However, regarding our data, there is a confirmation of an evident lack of access to specific information. However, despite the DGS guidelines for iodine supplementation in pregnant women [32], there is still an almost total absence of this supplementation and an awareness lack of knowledge [30].

WHO [46] defines food fortification as the practice of deliberately increasing the content of an essential micronutrient, i. e., vitamins and minerals (including trace elements) in food, in order to improve the nutritional quality of the food supply and provide a public health benefit with minimal health risk. Vitamin and mineral fortification and supplementation policies need to be promoted as the epidemiologic, nutritional, and sociological scientific basis of human nutrition expands, specifically addressing widespread deficiencies of micronutrients essential for individuals and population health. Therefore, all countries should adopt WHO recommendations, particularly addressing iodized salt in case of iodine supplementation for vulnerable groups, such as SAC [47]. The WHO recommends a daily intake of 90 μg of iodine for infants, 120 μg for schoolchildren (6–12 years), 150 μg for adolescents and adults and 200–300 μg for pregnant and lactating women [23, 47]. Currently, iodization levels of 20–40 mg iodine per Kg of salt are recommended [48]. Also EFSA [49] suggests dietary reference values for iodine daily intake.

In many countries, voluntary iodine fortification of salt is a common practice, but the contributions of iodine from this source are not reflected in the national food composition databases, except in Denmark [3]. Although regions where fruits, fresh vegetables and proteins are readily available and well represented in the average diet, nutritional deficiencies are supposed to be uncommon but wrong dietary decisions still lead to nutritional deficiencies. Dereby, in Switzerland, iodized salt (containing 20–30 mg I/Kg) has been available for decades. As a result, the incidence of goiter is now very low. On the contrary, in Germany, iodine deficiency is still present and a higher reference intake has been given [50]. According to Mensink et al. [3], the mean intake of iodine was generally lower for all aged groups in Germany and highest in Denmark. However, even countries with effective iodized salt programs will always have individuals classified with low iodine intakes based on the percentage of urinary iodine concentration UIC <100 μg/L [26, 27].

Also in United States, salt iodization remains one of the assured sources of iodine [51]. For the comproved efficiency, safety and also for low cost estimated (only 1.8 − 4.5 € per child covered per year) [27], this option is the best strategy to control iodine deficiency [52, 53]. Iodine at ppm levels in foods does not cause any sensory changes, and the price difference between iodized and non-iodized salt is negligible [53]. However, in most countries, Portugal included, there are no rules for labeling on food packing for iodine, making difficult to identify sources of iodine [54].

Cooking the food with iodized salt is a desirable practice because it guarantees the presence of this element. There are also other methods to provide iodine to the general population, such as adding iodine to drinking water or taking supplements of iodine [41]. That does not mean we should increase salt consumption. The suggestion is to use iodized salt instead of the normal salt.

## Conclusion

Study results reveal that the majority of children eat at school canteens, which is covered by the recommended use of iodized salt for school canteens. However, there are still no data to evaluate those recommendations are being followed. Also, there are no evidence to support the implementation of preventive measures issued by DGS and DGE, neither available data revealing iodine levels for general population and SAC in particular. Therefore, it was highlighted the lack of clarification regarding iodine knowledge and needs. It is suggested an urgent evaluation of iodine needs in Portugal and a possible iodized salt programme to be mandatory, namely for school-aged children where iodine deficiencies are critical, as well as a systematic information dissemination as a form of publicizing iodine supplementation needs.

## Additional file

Additional file 1: Survey questions. Questions on children’s diet and parental knowledge of iodine content used in this study. (PDF 155 kb)

## Abbreviations

DGE: Directorate-General for Education
DGS: Directorate-General for Health
SAC: School-age-children
WHO: World Health Organization

## References

[1] Boeing H, Oberritter H, Daniel H (2015). 12th European Nutrition Conference (FENS). Ann Nutr Metab.

[2] Blundell JE, Baker JL, Boyland E, Blaak E, Charzewska J, de Henauw S, Frühbeck G, Gonzalez-Gross M, Hebebrand J, Holm L, Kriaucioniene V, Lissner L, Oppert JM, Schindler K, Silva AM, Woodward E (2017). Variations in the Prevalence of Obesity Among European Countries, and a Consideration of Possible Causes. Obes Facts.

[3] Mensink GBM, Fletcher R, Gurinovic M, Huybrechts I, Lafay L, Serra-Majem L, Szponar L, Tetens I, Verkaik-Kloosterman J, Baka A, Stephen AM (2013). Mapping low intake of micronutrients across Europe. Br J Nutr.

[4] Serra-Majem L (2001). Vitamin and mineral intakes in European children. Is food fortification needed?. Public Health Nutr.

[5] Serra-Majem L, Ribas L, Pérez-Rodrigo C, García-Closas R, Peña-Quintana L, Aranceta J (2002). Determinants of Nutrient Intake among Children and Adolescents: Results from the enKid Study. Ann Nutr.

[6] Kaganov B, Caroli M, Mazur A, Singhal A, Vania A (2015). Suboptimal Micronutrient Intake among Children in Europe. Nutrients.

[7] Lazarus JH (2015). The importance of iodine in public health. Environ Geochem Health.

[8] EFSA NDA Panel (ESFA Panel on Dietetic Products, Nutrition and Allergies) (2013). Scientific Opinion on nutrient requirements and dietary intakes of infants and young children in the European Union. EFSA J.

[9] Viñas BR, Barba LR, Ngo J, Gurinovic M, Novakoric R, Cavelaars A, de Groot LCPGM, van’t Veer P, Matthys C, Serra-Majem L (2011). Projected Prevalence of Inadequate Nutrient Intakes in Europe. Ann Nutr Metab.

[10] Walker SP, Wachs TD, Gardner JM, Lozoff B, Wasserman GA, Pollitt E, Carter JA (2007). Child development: risk factors for adverse outcomes in developing countries. Lancet.

[11] Cavelaars AEJM, Doets EL, Dhonukshe-Rutten RAM, Hermoso M, Fairweather-Tait SJ, Koletzko B, Gurinović M, Moreno LA, Cetin I, Matthys C, van’t Veer P, Ashwell M, de Groot CPGM (2010). Prioritizing micronutrients for the purpose of reviewing their requirements: a protocol developed by EURRECA. Eur J Clin Nutr.

[12] Matthys C, Bucchini L, Busstra MC, Cavelaars AEJM, Eleftheriou P, Garcia-Alvarez A, Fairweather-Tait S, Gurinovic M, van Ommens B, Contor L (2010). EURRECA: development of tools to improve the alignment of micronutrient recommendations. Eur J Clin Nutr.

[13] World Health Organization (WHO) (2004). Global Database on Iodine Deficiency.

[14] World Health Organization (WHO) (1996). Iodine.

[15] Prashanth L, Kattapagari KK, Chitturi RT, Baddam VRR, Prasad LK (2015). A review on role of essential trace elements in health and disease. J Dr NTR Univ Health Sci.

[16] Miot F, Dupuy C, Dumont J, Rousset B. Thyroid hormone synthesis and secretion, NCBI Bookshelf. South Dartmouth (MA): National Library of Medicine, National Institutes of Health; 2015.

[17] Hauser P, Zametkin AJ, Martinez P, Vitiello B, Matochik JA, Mixson AJ, Weintraub BD (1993). Attention deficit-hyperactivity disorder in people with generalizes resistance to thyroid hormone. N Engl J Med.

[18] Vermiglio F, Lo Presti VP, Moleti M, Sidoti M, Tortorella G, Scaffidi G, Castagna MG, Mattina F, Violi MA, Crisà A, Artemisia A, Trimarchi F (2004). Attention Deficit and Hyperactivity Disorders in the Offspring of Mothers Exposed to Mild-Moderate Iodine Deficiency: A Possible Novel Iodine Deficiency Disorder in Developed Countries. J Clin Endocrinol Metabol.

[19] Qian M, Wang D, Watkins WE, Gebski V, Yan YQ, Li M, Chen ZP (2005). The effects of iodine on intelligence in children: A meta-analysis of studies conducted in China. Asia Pac J Clin Nutr.

[20] Delange F (2001). Iodine deficiency as a cause of brain damage. Postgrad Med J.

[21] Santiago-Fernandez P, Torres-Barahona R, Muela-Martínez JÁ, Rojo-Martinéz G, García-Fuentes E, Garriga MJ, León AG, Soriguer F (2004). Intelligence Quotient and Iodine Intake: A Cross-Sectional Study in Children. J Clin Endocrinol Metabol.

[22] EFSA NDA Panel (ESFA Panel on Dietetic Products, Nutrition and Allergies) (2014). Scientific Opinion on the substantiation of a health claim related to iodine and contribution to normal cognitive development pursuant to Article 14 of Regulation (EC) N°. 1924/20061. EFSA J.

[23] World Health Organization (WHO) (2007). Assessment of iodine deficiency disorders and monitoring their elimination.

[24] World Health Organization (WHO) (2003). Diet, Nutrition, and the Prevention of Chronic Diseases, Joint WHO/FAO Technical Report Series.

[25] Andersson M, Takkouche B, Egli I, Allen HE, de Benoit B (2005). Current global iodine status and progress over the last decade towards the elimination of iodine deficiency. Bull World Health Organ.

[26] Andersson M, Karumbunathan V, Zimmermann MB (2012). Global Iodine Status in 2011 and Trends over the Past Decade. J Nutr.

[27] Zimmermann MB, Andersson M (2012). Update on iodine status worldwide. Curr Opin Endocrinol Diabetes Obes.

[28] Vanderpump MPJ, Lazarus JH, Smyth PP, Laurberg P, Holder RL, Boelaert K, Franklyn JA (2011). Iodine status of UK schoolgirls: a cross-sectional survey. Lancet.

[29] UNICEF (2004). The State of the World’s Children 2005: childhood under threat.

[30] Costeira MJ, Oliveira P, Ares S, de Escobar GM, Palha JA (2009). Iodine status of pregnant women and their progeny in the Minho region of Portugal. Thyroid.

[31] Limbert E, Prazeres S, São Pedro M, Madureira D, Miranda A, Ribeiro M, Jácome de Castro J, Carrilho F, Oliveira MJ, Reguengo H, Borges F, Tyroid Study Group of the Portuguese Endocrine Society (2010). Iodine intake in Portuguese pregnant women: results of a countrywide study. Eur J Endocrinol.

[32] Direção-Geral da Saúde (DGS) (2013). Orientação da Direção-Geral da Saúde.

[33] Jacob M, Brito N (2015). Suplementação de iodo na gravidez: qual a importância?. Rev Port Saúde Pública.

[34] Delange F, Bürgi H, Chen ZP, Dunn JT (2002). World Status of Monitoring of Iodine Deficiency Disorders Control Programs. Tryroid.

[35] Limbert E, Prazeres S, São Pedro M, Madureira D, Miranda A, Ribeiro M, Carrilho F, Jácome de Castro J, Santana Lopes M, Cardoso J, Carvalho A, Oliveira MJ, Reguengo H, Borges F, Grupo de Estudos da Tiróide da Sociedade Portuguesa de Endocrinologia, Diabetes e Metabolismo (2012). Aporte do Iodo nas Crianças das Escolas em Portugal. Acta Medica Port.

[36] Limbert E, Prazeres S, Madureira D, Miranda A, Ribeiro M, Abreu FS, César R, Ferreira AM, Ferreira M, Sá M, Lemos L, Carvalho R, Ponte C, Mota L, Carrilho F, Jácome de Castro J, Oliveira MJ, Grupo de Estudos da Tiróide da Sociedade Portuguesa de Endocrinologia, Diabetes e Metabolismo (2012). Aporte do Iodo nas Crianças das Escolas em Portugal. Rev Port Endocrinol Diab Metab.

[37] Andrews D, Nonnecke B, Preece J (2003). Electronic survey methodology: A case study in reaching hard to involve Internet Users. Int J Hum Comput Interact.

[38] Nulty DD (2008). The adequacy of response rates to online and paper surveys: what can be done?. Assess Eval High Educ.

[39] Wright KB. Researching Internet-Based Populations: Advantages and Disadvantages of Online Survey Research, Online Questionnaire Authoring Software Packages, and Web Survey Services. J Comput-Mediat Commun. 2005;10. doi:10.1111/j.1083-6101.2005.tb00259.x. Available on: http://onlinelibrary.wiley.com/doi/10.1111/j.1083-6101.2005.tb00259.x/full.

[40] Roman-Viñas B, Barba RL, Ngo J, Gurinovic M, Novakovic R, Cavelaars A, de Groot LCPGM, van’t Veer P, Matthys C, Serra-Majem L (2011). Projected prevalence of inadequate nutrient intakes in Europe. Ann Nutr Metab.

[41] Santana Lopes M, Jácome de Castro J, Marcelino M, Oliveira MJ, Carrilho F, Limbert E, de Estudos da Tiróide G (2012). Iodo e Tiróide: O que o Clínico deve Saber. Acta Medica Port.

[42] Haldimann M, Alt A, Blanc A, Blondeau K (2005). Iodine content of food groups. J Food Compos Anal.

[43] Zimmermann MB, Andersson M (2012). Assessment of iodine nutrition in populations: past, present, and future. Nutr Rev.

[44] Direção-Geral da Educação (DGE) (2013). Circular: Orientações sobre ementas e refeitórios escolares – 2013/2014.

[45] Taylor PN, Okosieme OE, Dayan CM, Lazarus JH (2014). Impact of iodine supplementation in mild-to-moderate iodine deficiency: systematic review and meta-analysis. Eur J Endocrinol.

[46] World Health Organization (WHO) (2006). Guidelines on food fortification with micronutrients.

[47] Tulchinsky TH (2010). Micronutrient Deficiency Conditions: Global Health Issues. Public Health Rev.

[48] Wu T, Liu GJ, Li P, Clar C (2002). Iodized salt for preventing iodine deficiency disorders. Cochrane Database Syst Rev.

[49] EFSA NDA Panel (ESFA Panel on Dietetic Products, Nutrition and Allergies) (2014). Scientific Opinion on dietary reference values for iodine. EFSA J.

[50] Prentice A, Branca F, Decsi T, Michaelsen KF, Fletcher RJ, Guesry P, Manz F, Vidailhet M, Pannemans D, Samartín S (2004). Energy and nutrient dietary reference values for children in Europe: methodological approaches and current nutritional recommendations. Br J Nutr.

[51] Dasgupta PK, Liu Y, Dyke JV (2007). Iodine Nutrition: Iodine Content of Iodized Salt in the United States. Environ Sci Technol.

[52] Zimmermann MB (2009). Iodine Deficiency. Endocr Rev.

[53] Pearce EN, Andersson M, Zimmermann MB (2013). Global iodine nutrition: Where do we stand in 2013?. Thyroid.

[54] Teixeira D, Calhau C, Pestana D, Vicente L, Graça P. IODO – Importância para a saúde e o papel da alimentação, Programa Nacional para a Promoção da Alimentação Saudável: DGS; 2014. Available on: https://www.alimentacaosaudavel.dgs.pt/activeapp/wpcontent/files_mf/1444899433Iodo_Import%C3%A2nciaparaasa%C3%BAdeeopapeldaalimenta%C3%A7%C3%A3o.pdf.

