# Supplementary material for: Assessment of iodine importance and needs for supplementation in school-aged children in Portugal
Source: BMC Nutr. 2017 Jul 20;3:64. doi: 10.1186/s40795-017-0175-x (PMC7050902; doi:10.1186/s40795-017-0175-x)
Supplement: Supplementary file 1 — Survey questions. Questions on children’s diet and parental knowledge of iodine content used in this study. (PDF 155 kb) [file 40795_2017_175_MOESM1_ESM.pdf]

Answer to your child's food frequency survey for the following foods: *

Mark only one option per line

	2 or more times a day	1 time per day	5 to 6 times a week	2 to 4 times a week	1 time a week	1 to 3 times a month	Rarely (less than 1 time per month)	Never
Meat								
Fish								
Seafood								
Eggs								
Bread/Cereals								
Milk								
Yogurts								
Cheese								
Fruits								
Vegetables								
Legumes (beans, chickpeas, peas, ...)								
Algae								

Iodine is found in the following foods: *

Mark only one option per line

	Yes	No	Do not know
Meat			
Seafood			
Eggs			
Bread/Cereals			
Milk			
Yogurts			
Cheese			
Fruits			
Vegetables			
Legumes (beans, chickpeas, peas, ...)			
Algae			
